# Shining a light on autophagy in neurodegenerative diseases

**DOI:** 10.1016/j.jbc.2021.101437

**Published:** 2021-11-19

**Authors:** Aswathy Chandran, Jean-Christophe Rochet

**Affiliations:** 1 Department of Medicinal Chemistry and Molecular Pharmacology, Purdue University, West Lafayette, Indiana, USA; 2 Purdue Institute for Integrative Neuroscience, Purdue University, West Lafayette, Indiana, USA

**Keywords:** amyotrophic lateral sclerosis, autophagic flux, autophagosome, autophagy, Dendra2, LC3, lysosome, neurodegenerative disease, protein aggregate, screening, AD, Alzheimer’s disease, ALS, amyotrophic lateral sclerosis, CMA, chaperone-mediated autophagy, FTD, frontotemporal dementia, PD, Parkinson’s disease, UPS, ubiquitin proteasome system

## Abstract

Small-molecule modulators of autophagy have been widely investigated as potential therapies for neurodegenerative diseases. In a recent issue of JBC, Safren *et al.* described a novel assay that uses a photoconvertible fusion protein to identify compounds that alter autophagic flux. Autophagy inducers identified using this assay were found to either alleviate or exacerbate neurotoxicity in different cellular models of amyotrophic lateral sclerosis, challenging the notion that autophagy stimulation can be used as a one-size-fits-all therapy for neurodegenerative disease.

Cellular proteostasis is governed by a tight coordination between protein synthesis and protein elimination. The latter process involves protein degradation by either the ubiquitin proteasome system (UPS) or the autophagy-lysosome pathway (ALP), encompassing macroautophagy, microautophagy, and chaperone-mediated autophagy (CMA). Multiple lines of evidence suggest that macroautophagy (referred to hereafter as “autophagy”) plays a key role in eliminating toxic protein aggregates frequently observed in neurodegenerative diseases, in turn implying that upregulation of autophagy could be neuroprotective. However, progress in identifying small molecules that induce autophagy has been limited by the drawbacks of existing assays designed to monitor “autophagic flux,” a term used to describe the entire dynamic process of autophagic degradation.

Autophagy involves the encapsulation of cellular components (or “cargo”) into double-membraned structures named autophagosomes. Autophagosomes subsequently fuse with lysosomes, resulting in the degradation of the cargo by lysosomal enzymes. The initiation of autophagosome formation is regulated by numerous upstream signaling proteins, including the kinases mTOR and AMPK. The pathology of neurodegenerative disorders such as Alzheimer’s disease (AD), Parkinson’s disease (PD), amyotrophic lateral sclerosis (ALS), and frontotemporal dementia (FTD) involves an accumulation in the central nervous system (CNS) of fibrillar aggregates formed by one or more peptides or proteins, including amyloid-beta and tau in AD, alpha-synuclein in PD, and TDP-43 and FUS in ALS/FTD. Autophagy is thought to be primarily responsible for eliminating these oligomers or fibrils in the brains of neurodegenerative disease patients, as the UPS is incapable of degrading such large protein aggregates (1, 2). Autophagic dysfunction is an important pathologic feature of neurodegenerative diseases (3). Mutations in several genes associated with different stages of the autophagy pathway have been identified as risk factors for the development of familial neurodegenerative diseases. Autophagic dysfunction is likely also a factor in idiopathic neurodegenerative diseases, where the presence of protein aggregates impairs autophagic degradation in the absence of any underlying mutations (4).

Recently, substantial research efforts have been focused on developing strategies to boost the autophagic clearance of protein aggregates for therapeutic purposes in neurodegenerative disorders (5). Conventional screens aimed at identifying small-molecule autophagy activators typically involve cellular assays to monitor the steady-state levels of LC3 (a protein that attaches to the autophagosome membrane and is subsequently degraded with the cargo) fused to a fluorescent protein such as GFP. However, data from these steady-state assays are generally difficult to interpret because upregulation of LC3-GFP can reflect increased autophagosome formation or impaired autophagosome–lysosome fusion at the upstream and downstream ends of the pathway, respectively. Therefore, hits from chemical screens carried out using this method must be validated with orthogonal assays designed to compare rates of autophagic substrate turnover in cells incubated with or without lysosomal inhibitors, an approach that can yield confounding results due to inhibitor cytotoxicity (6).

To address these challenges, Safren *et al.* (7) developed a novel, comprehensive, multilevel screening assay to identify autophagy modulators by expressing LC3 fused to a photoconvertible variant of GFP named Dendra2 (Fig. 1*A*). Dendra2-LC3 normally emits green fluorescence, but can be photoconverted to emit red fluorescence upon exposure to UV light. This strategy enables labeling of a specific pool of cellular Dendra2-LC3 that can be tracked over time *via* measurements of red fluorescence to monitor autophagic flux. The authors generated a stable HEK293T cell line using CRISPR/Cas9-mediated gene editing to add the Dendra2 module to endogenous LC3, thereby avoiding artifacts arising from LC3 overexpression. Additionally, using Dendra2 to label endogenous LC3 enabled the authors to monitor the green fluorescence emitted by Dendra2-LC3 as a primary endpoint to identify potential autophagy inducers. The hits obtained from an initial round of screening were reexamined by measuring autophagic flux after photoconversion and further validated using previously established assays, including immunoblot analysis of LC3 levels and measurements of intracellular GFP-RFP-LC3 fluorescence. Importantly, multiple compounds previously identified as autophagy inducers *via* measurements of steady-state LC3 levels failed to show the same stimulatory effect in the Dendra2-LC3 cell line, highlighting the potential risk of false positives inherent in existing steady-state assays.

**Figure 1:**
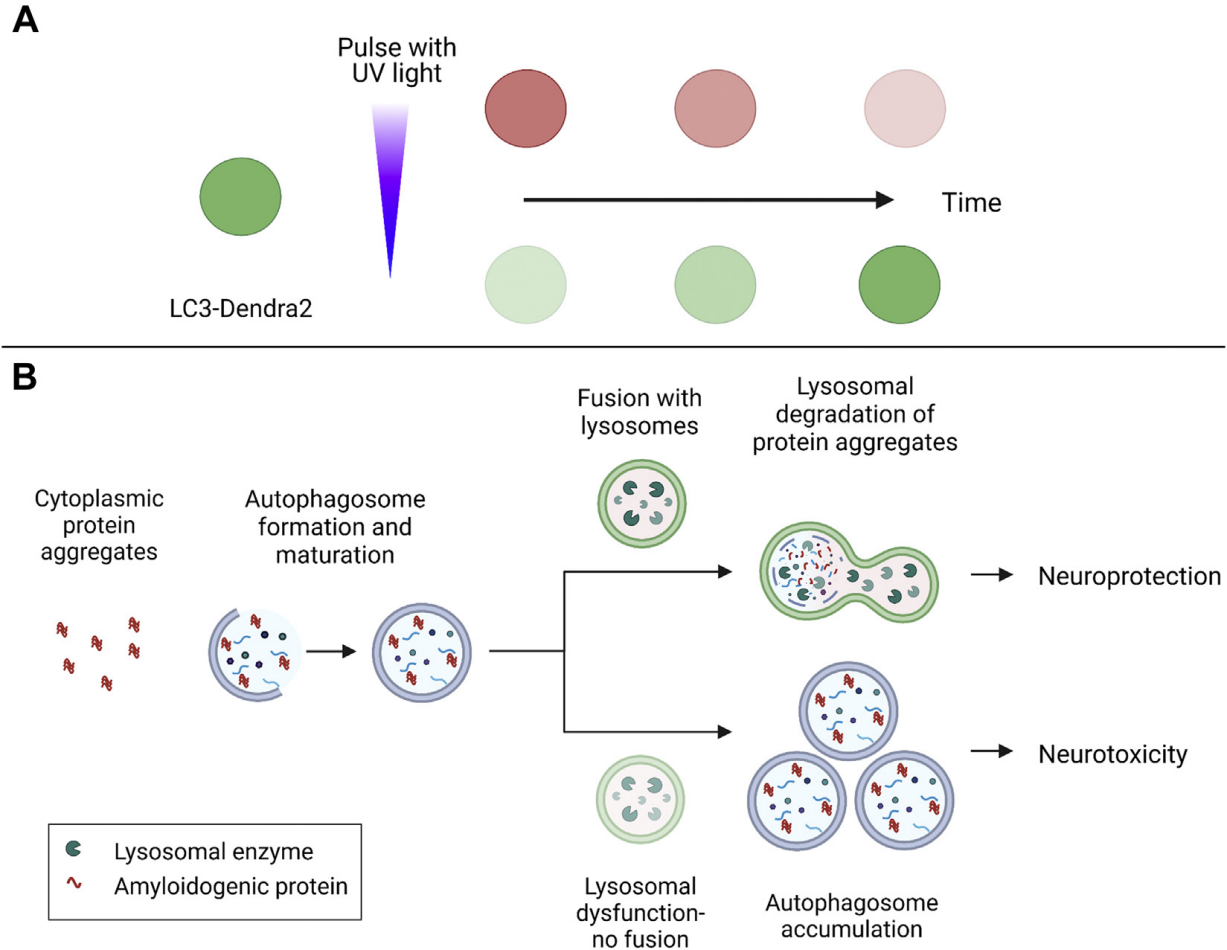
**The neuroprotective potential of autophagy inducers depends on the efficiency of autophagic flux.***A*, schematic of the experimental design used by Safren *et al.* to identify autophagy modulators. Dendra2-LC3 undergoes photoconversion, resulting in a switch from *green* to *red* fluorescence, in cells exposed to UV light. Red Dendra2-LC3 can then be imaged over time to monitor autophagic flux, whereas the green fluorescent protein can be imaged to monitor autophagosome formation. *B*, schematic illustrating different outcomes of autophagy induction depending on the status of autophagic flux. Cytoplasmic protein aggregates can be engulfed by an autophagosome that subsequently fuses with a lysosome, resulting in aggregate degradation and alleviation of neurotoxicity (*upper pathway*). In cells with downstream autophagic impairment (*e.g.*, because of lysosomal defects that preclude autophagosome-lysosome fusion), autophagy induction results in a buildup of autophagosomes and exacerbation of neurotoxicity (*lower pathway*). Created with BioRender.com.

A subset of autophagy inducers identified using the Dendra2-LC3 assay were tested for potential neuroprotective effects in cultured neurons expressing different ALS- or ALS/FTD-related proteins. Based on evidence that autophagic impairment plays a role in neurodegeneration, autophagy upregulation would be predicted to alleviate neurotoxicity by stimulating the clearance of protein aggregates, whereas autophagy inhibitors should have the opposite effect (8). Consistent with this idea, the authors found that autophagy inducers enhanced the survival of neurons overexpressing TDP-43. Surprisingly, however, opposing effects were observed in neurons expressing mutant forms of UBQLN2, a receptor protein involved in delivering ubiquitinated proteins to proteasomes, and in neurons harboring an ALS/FTD-linked *C9ORF72* mutation.

An important outcome of this study is the demonstration that the nature of the disease-related autophagic impairment dictates whether activating autophagy can lead to neuroprotection (Fig. 1*B*). Because TDP-43 overexpression leads to the formation of neurotoxic, cytoplasmic aggregates, autophagy induction is expected to increase TDP-43 turnover by promoting the clearance of TDP-43 aggregates. In contrast, mutant UBQLN2 has been shown to modulate autophagy directly by impairing lysosome acidification (9), and *C9ORF72* gene knockouts are linked to defective endolysosomal function (10). Induction of autophagy in such cases likely leads to a toxic accumulation of autophagosomes that cannot be eliminated by fusion with lysosomes in neurons expressing mutant UBQLN2 or carrying the *C9ORF72* mutation. Therefore, in these cases, autophagy inducers must be combined with agents that stimulate lysosomal clearance to achieve a neuroprotective effect.

The Dendra2-LC3 platform developed by Safren *et al.* (7) has the potential to open new research avenues aimed at unraveling proteostasis mechanisms—for example, by coexpressing the Dendra2-LC3 biosensor with photoconvertible UPS or CMA reporters to examine the interplay among different clearance systems. The systematic screening pipeline described in this report will advance efforts to identify autophagy modulators of therapeutic benefit in neurodegenerative disorders as well as in other diseases, including cancer. Finally, the authors’ finding that autophagy inducers fail to exert a neuroprotective effect in neurons with defective lysosomal proteolysis sets the stage for developing tailored therapies (*e.g.*, an autophagy inducer coupled with a lysosomal clearance enhancer) to account for variations in autophagic flux among different neurodegenerative diseases or disease subtypes.

## Conflict of interest

The authors declare that they have no conflicts of interest with the contents of this article.
